# scMMT: a multi-use deep learning approach for cell annotation, protein prediction and embedding in single-cell RNA-seq data

**DOI:** 10.1093/bib/bbad523

**Published:** 2024-01-31

**Authors:** Songqi Zhou, Yang Li, Wenyuan Wu, Li Li

**Affiliations:** Chongqing Institute of Green and Intelligent Technology, Chinese Academy of Sciences, Chongqing, China; Chongqing School, University of Chinese Academy of Sciences, Chongqing, China; Chongqing Institute of Green and Intelligent Technology, Chinese Academy of Sciences, Chongqing, China; Chongqing School, University of Chinese Academy of Sciences, Chongqing, China; Chongqing Research Institute of Big Data, Peking University, Chongqing, China; Chongqing Institute of Green and Intelligent Technology, Chinese Academy of Sciences, Chongqing, China; Chongqing School, University of Chinese Academy of Sciences, Chongqing, China; Chongqing Institute of Green and Intelligent Technology, Chinese Academy of Sciences, Chongqing, China; Chongqing School, University of Chinese Academy of Sciences, Chongqing, China

**Keywords:** multi-task learning, scRNA-seq, cell annotation, protein prediction, low-dimensional embedding

## Abstract

Accurate cell type annotation in single-cell RNA-sequencing data is essential for advancing biological and medical research, particularly in understanding disease progression and tumor microenvironments. However, existing methods are constrained by single feature extraction approaches, lack of adaptability to immune cell types with similar molecular profiles but distinct functions and a failure to account for the impact of cell label noise on model accuracy, all of which compromise the precision of annotation. To address these challenges, we developed a supervised approach called scMMT. We proposed a novel feature extraction technique to uncover more valuable information. Additionally, we constructed a multi-task learning framework based on the GradNorm method to enhance the recognition of challenging immune cells and reduce the impact of label noise by facilitating mutual reinforcement between cell type annotation and protein prediction tasks. Furthermore, we introduced logarithmic weighting and label smoothing mechanisms to enhance the recognition ability of rare cell types and prevent model overconfidence. Through comprehensive evaluations on multiple public datasets, scMMT has demonstrated state-of-the-art performance in various aspects including cell type annotation, rare cell identification, dropout and label noise resistance, protein expression prediction and low-dimensional embedding representation.

## INTRODUCTION

Conventional bulk sequencing methods often overlook the inherent cellular heterogeneity, whereas the advent of single-cell RNA-sequencing (scRNA-seq) technology has revolutionized the field of genomics by enabling the identification and characterization of cellular differences at the individual cell level [1]. This powerful technique has found widespread applications in various disciplines, including developmental biology, immunology, neuroscience and oncology [2–4]. Accurate annotation of cell types in scRNA-seq data is crucial for gaining a comprehensive understanding of the cellular heterogeneity within complex tissues and organs. This understanding is essential for advancing biological and medical research, particularly in the context of elucidating disease progression and tumor microenvironments [5]. Given the highly challenging nature of accurately annotating single-cell data, the development of increasingly accurate automated cell annotation methods has provided valuable assistance for the rapid advancement of this field [6–8].

With the continuous advancement of sequencing technology, such as the emergence of the multi-modal sequencing technique [9, 10], and the increasing need for higher precision in identifying cell types, existing methods have revealed some aspects that require further improvement. Firstly, current methods [11–16] often struggle to extract valuable information and eliminate noise from non-highly variable genes (non-HVGs), leading to an overreliance on highly variable genes (HVGs) alone. However, non-HVGs may contain important biological insights, and relying solely on HVGs could limit the comprehensive utilization of scRNA-seq data [17]. Moreover, the current methods that solely rely on scRNA-seq data often struggle to distinguish between immune cell categories with similar molecular profiles but distinct functions, despite the considerable functional diversity among these cell types [18, 19]. Additionally, cell type distribution within biological systems typically exhibits inherent unevenness, coupled with the possibility of manual annotation errors [20, 21], presenting significant challenges in predicting cell types, particularly for rare cell types. These challenges limit the capacity of automated cell annotation tools, necessitating the development of a new method with improved recognition capabilities.

To fully leverage the abundant information present in scRNA-seq data, we have developed a novel deep learning approach for cell annotation, protein expression prediction and low-dimensional embedding, which we named ‘single-cell multi-modal data and multi-task learning tool’ (scMMT). Due to the selection of HVGs based on their expression variance across the entire dataset, with predominant cell types contributing the most variance, there is a risk of overlooking crucial genes in rare cell types and neglecting the co-occurrence and biological interactions of genes, particularly between HVGs and non-HVGs [8, 22]. We employed a novel combination of multiple methods to extract information from scRNA-seq data, enabling a more comprehensive utilization of the data.

Furthermore, an increasing number of multi-modal and multi-task learning methods [23] have been utilized across various domains and achieved superior performance [24–26]. It’s important to recognize that different single-cell analysis tasks are also interconnected, such as cells of the same type generally exhibit similar protein expression levels [18, 19], establishing a relationship between cell type annotation and protein prediction tasks. Multi-modal sequencing technologies, such as CITE-seq [9], enabling simultaneous measurement of both RNA and surface proteins in cells, provide an opportunity for multi-task learning. Hence, we used scRNA-seq data as input and protein expression levels and cell types as outputs to construct a multi-task learning framework based on the GradNorm [27] method. Indeed, proteins are more abundant than RNA and play direct roles in cellular signaling and intercellular interactions [28, 29]. Consequently, integrating protein information into multi-task learning can offer supplementary insights. This not only has the potential to address the constraints of scRNA-seq in distinguishing immune cell types with similar RNA profiles but distinct functions [7, 30, 31] but also mitigates the impact of noise in cell type labels. Additionally, we introduced logarithmic weighting and label smoothing mechanisms to enhance the recognition ability of rare cell types and prevent model overconfidence.

Through extensive evaluations on multiple public datasets, scMMT has consistently shown exceptional performance in various aspects, such as cell type annotation, rare cell identification, label noise resistance, protein expression prediction and low-dimensional embedding representation. As the size of public multi-modal datasets continues to expand, scMMT and its innovative approaches to data processing hold great promise for advancing research in the field of single-cell genomics.

## MATERIALS AND METHODS

### Dataset preparation

To ensure a more persuasive comparison with mainstream methods, we selected the most popular Seurat-v4 human peripheral blood mononuclear cells dataset [7] (160k PBMCs dataset) for our main analysis and maintained consistency with the mainstream methods in the partitioning of the training and validation sets. Additionally, in order to assess the generalization and transferability of our model, we also selected the Haniffa COVID-19 dataset [32] (COVID-19 dataset) and H1N1 influenza PBMCs dataset [31, 33] (H1N1 dataset) to evaluate relevant tasks. These choices were based on the challenging nature of immune cell. Importantly, these datasets were sourced from authoritative institutions, encompassing a substantial number of cells and ensuring reliable data quality and labels.

To assess the model’s robustness to dropout noise in scRNA-seq data, we created a CITE-seq simulation dataset based on the 160k PBMCs dataset. Specifically, we non-repetitively sampled the four most abundant cell types (‘CD4’, ‘CD8’, ‘NK’, ‘Mono’) from donor 1’s data, extracting 5000 cells from each type to create the new CITE-seq simulation dataset. This approach effectively mitigates the impact of batch effects and unbalanced labels on the RNA and protein data while preserving their intrinsic relationship. Therefore, the continuous integration of dropout noise provides a more intuitive assessment of the model’s robustness when applied to this dataset.

These datasets and their respective partitions in each experiment are detailed in Table 1 and Supplementary Table S1 available online at http://bib.oxfordjournals.org/, respectively.

**Table 1:** Summary of datasets

Dataset	Batches	Cells	Genes	Proteins	Cell types
160k PBMCs dataset	8	161 764	20 729	224	8/31/58
H1N1 dataset	20	53 201	32 738	87	#
COVID-19 dataset	143	647 366	24 737	192	50
Simulation dataset	1	20 000	20 729	224	4

### Overview of scMMT

The main objective of scMMT is to maximize the value of scRNA-seq query datasets using CITE-seq datasets as reference datasets. Our model was trained using a censored loss function approach [34] which focused on measured proteins and censored unmeasured ones in each cell. This enabled the integration of multiple CITE-seq datasets, even when they had different protein panels. Notably, scMMT has the flexibility to utilize scRNA-seq datasets as alternative references, albeit with a slight decrease in performance. This adaptability provides scMMT with a wider range of applications than other multi-modal methods [7, 34, 35].

**Figure 1:**
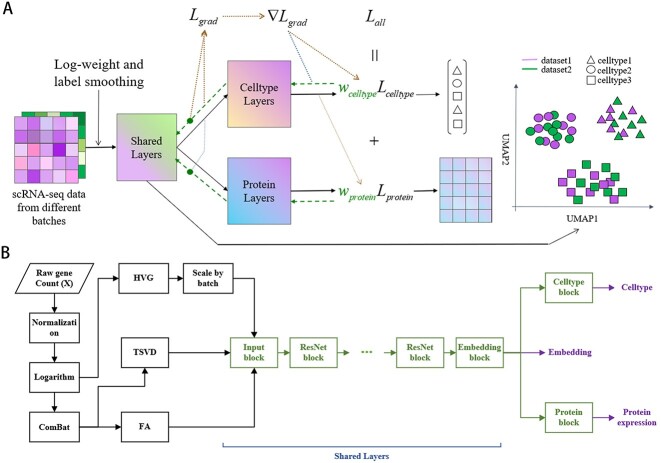
Overview of scMMT. (**A**) scMMT is a flexible multi-task learning framework that is capable of simultaneously performing multiple tasks, including cell type annotation, protein expression prediction and low-dimensional embedding. To enhance the applicability and generalization capabilities of the model, we have incorporated several mechanisms, including logarithmic weighting and label smoothing. Furthermore, we have utilized the GradNorm gradient loss to balance the gradients across different tasks, thereby improving the performance of various tasks in the model. (**B**) scMMT model comprises a range of modules that are designed to extract features, process inputs and output predictions. The data preprocessing section, represented by the black boxes, includes steps such as normalization, logarithm transformation, ComBat, HVG, scaling, TSVD and FA. The neural network structure, represented by the green boxes, consists of an input block, a series of ResNet blocks, a low-dimensional embedding block and two single task blocks that predict cell types and protein expression levels respectively, as indicated by the purple letters.

Figure 1A illustrated the construction of a multi-task learning model comprising shared, cell type prediction and protein expression prediction layers. These modules generated low-dimensional embeddings for both the reference and query datasets as well as the predicted cell annotations and protein expressions for the query dataset. To address the issue of imbalanced samples in the reference dataset, we designed a logarithmic weighting method that moderately increased the weight of rare cell types. Moreover, to mitigate potential errors in the manually annotated cell types, we incorporated a label smoothing mechanism to prevent the neural network model from becoming overly confident [36]. During backpropagation, we employed the GradNorm algorithm to dynamically adjust the gradient magnitudes for both cell annotation and protein expression prediction tasks, which allowed the two tasks to reinforce each other mutually and effectively reduced overfitting.

Unlike existing methods, we employed a combination of HVGs, truncated singular value decomposition (TSVD) [37] and factor analysis (FA) [38] to extract features from scRNA-seq data (Figure 1B). The advantage of TSVD lies in its ability to explain all the variance of the original variables, thereby compensating for the limitation of HVGs in effectively extracting information from non-HVGs, and it can also eliminate noise and redundant information in the data. On the other hand, FA primarily aims to describe the interdependencies within the correlation matrix of the original variables, portraying the relationships among genes and addressing the problem of HVGs in neglecting the co-occurrence and biological interactions of genes. Each method offered a unique perspective on the data, allowing us to capture diverse aspects of biological variability and intracellular relationships by combining their results. However, due to technological variations, significant batch effects exist between different datasets. Placing exclusive reliance on features extracted solely through TSVD and FA could result in the model excessively depending on the distribution of the training set. This could lead to the presence of batch effects when making predictions across different datasets. To address this issue, we adopted ComBat [39] method for data integration before applying TSVD and FA methods, which effectively reduced batch differences across diverse datasets and enhanced the transferability of the model [40]. Furthermore, to address the problems of gradient vanishing and explosion during training and accelerate network convergence, we incorporated the ResNet [41] block as the fundamental unit of our neural network structure. By leveraging this architecture, scMMT achieved improved training efficiency and performance.

### Data preprocessing

We illustrated the workflow by using CITE-seq datasets as reference datasets and an scRNA-seq dataset as a query dataset. If scRNA-seq datasets are used as reference datasets, the protein counts can be set to zero. In the embedding task, if there is no query dataset, it can be left empty.

Let $k$ CITE-seq datasets be available for integration with a possible query scRNA-seq data to predict proteins and annotate cell types. Each CITE-seq dataset (indexed as $i$) consisted of an ${n}_i\times{g}_i$ RNA array and an ${n}_i\times{p}_i$ protein array. As we employed the censored loss function, the $k$ CITE-seq datasets can be merged to form an ${n}_{\mathrm{ref}}\times{g}_{\mathrm{ref}}$ RNA array and an ${n}_{\mathrm{ref}}\times{p}_{\mathrm{ref}}$ protein array. The dimensions of RNA and protein are calculated as follows:


(1) \begin{equation*} {g}_{\mathrm{ref}}={g}_1\cap{g}_2\cdots \cap{g}_k \end{equation*}

(2) \begin{equation*} {p}_{\mathrm{ref}}={p}_1\cup{p}_2\cdots \cup{p}_k \end{equation*}

The scRNA-seq dataset under query comprised a ${n}_q\times{g}_q$ RNA array. Then, we screened out genes that overlap between reference datasets and the query dataset as ${g}^{\prime }={g}_{\mathrm{ref}}\cap{g}_q$ and replaced the previous genes with the overlapping genes as the final input dimension.

Next, certain filtering steps were applied to remove cells and genes with low expression. By default, the program removes cells expressing fewer than 200 genes and genes expressing fewer than 30 cells. However, in this study, to ensure that all query sequences receive annotation and maintain consistency with other methods, no filtering was applied to any datasets.

### Feature extraction

Firstly, we employed normalization and logarithmic transformation on the RNA array, similar to conventional methods. This was done to ensure that the data were standardized and mitigate the impact of any potential outliers. Then, we proceed to extract features from two different perspectives. On the one hand, we employed a conventional approach to select 550 HVGs and performed standardization. On the other hand, we utilized the ComBat method to mitigate batch effects, followed by the implementation of TSVD and FA methods to extract 300 and 180 features, respectively. Then, these features were combined together as the final input features. Here, we have opted for a lower-dimensional parameter as the default setting to accommodate the prevailing scenarios. However, in cases where the primary focus is on major cell types or where the marker genes of interest are predominantly ranked high among the HVGs, expanding the selection of HVGs can yield favorable results. Conversely, in scenarios where the emphasis is on rare cell types or where the marker genes of interest are primarily non-HVGs due to factors such as dropout noise, appropriately increasing the dimensions of TSVD and FA can enhance the capture of more informative features.

### Model architecture

The neural network architecture of scMMT is depicted by the green box in Figure 1B. The specific structures of the blocks included Input block, ResNet block and Embedding block (Figure 1B, Supplementary Figure S1 available online at http://bib.oxfordjournals.org/). Additionally, two single-task blocks, each comprising a linear layer, are incorporated. The purposes of these blocks were to separately predict the cell type and protein expression. By employing separate single-task blocks, the model was capable of learning distinct representations and making independent predictions for each task. This flexibility allowed for addressing different biological questions and provided insights into both cell type annotation and protein expression prediction. The integration of these blocks into our experimental setup notably improved the overall performance and accuracy of our analysis. Specifically, we utilized four ResNet blocks to conduct our experimental analysis in this study. In the tasks of cell type prediction and protein expression prediction, we employed label smoothing cross-entropy [36] and mean squared error as the respective loss functions.

### Logarithmic weighting

To address the issue of imbalanced sample sizes across cell types, we applied logarithmic weighting to assign higher weights to cell types with fewer samples, thereby giving them more importance during the training process. The weighting function is defined as follows:


(3) \begin{equation*} \mathrm{weight}=\mathit{\log}\left(\frac{\sum{\mathrm{count}}_i}{{\mathrm{count}}_{\mathrm{i}}}+\alpha \right),\alpha >0 \end{equation*}

where count represents the number of samples for each cell type.

### GradNorm method

The formula for GradNorm, which we used to balance gradients during the backpropagation process for cell type annotation and protein prediction tasks, is as follows:


(4) \begin{equation*} {L}_{\mathrm{grad}}\left(t;{w}_i(t)\right)=\sum_i{\left|{G}_W^{(i)}(t)-{\overline{G}}_W(t)\times{\left[{r}_i(t)\right]}^{\alpha}\right|}_1 \end{equation*}

where ${G}_W^{(i)}(t)={\left\Vert{\nabla}_W{w}_i(t){L}_i(t)\right\Vert}_2$, represents the ${L}_2$ norm of gradient of the weighted single-task loss ${w}_i(t){L}_i(t)$ with respect to the chosen weights $W$; ${\overline{G}}_W(t)={E}_{\mathrm{task}}[{G}_W^{(i)}(t)]$ represents the average gradient norm across all tasks at training time $t$. ${\tilde{L}}_i(t)={L}_i(t)/{L}_i(0)$, represents the loss ratio for task $i$ at time $t$; ${r}_i(t)={\tilde{L}}_i(t)/{E}_{\mathrm{task}}[{\tilde{L}}_i(t)]$ represents the relative inverse training rate of task $i$. The incorporation of this method has led to increased stability and consistency during the training phase across the complete model, resulting in improved performance in each task.

## RESULTS

### scMMT enables more accurate cell type annotation

We conducted a comparative analysis of cell type annotation using two datasets: the 160k PBMCs dataset and the COVID-19 dataset. Notably, the 160k PBMCs dataset provided three levels of cell type annotation, enabling us to assess the impact of the number of annotated cell types on the accuracy. We evaluated the effectiveness of nine methods [8, 34, 42–48] along with our proposed methods: scMMT, which utilized CITE-seq datasets as reference datasets, and scMMT_RNA, which utilized scRNA-seq datasets as reference datasets and can only predict cell type.

**Figure 2:**
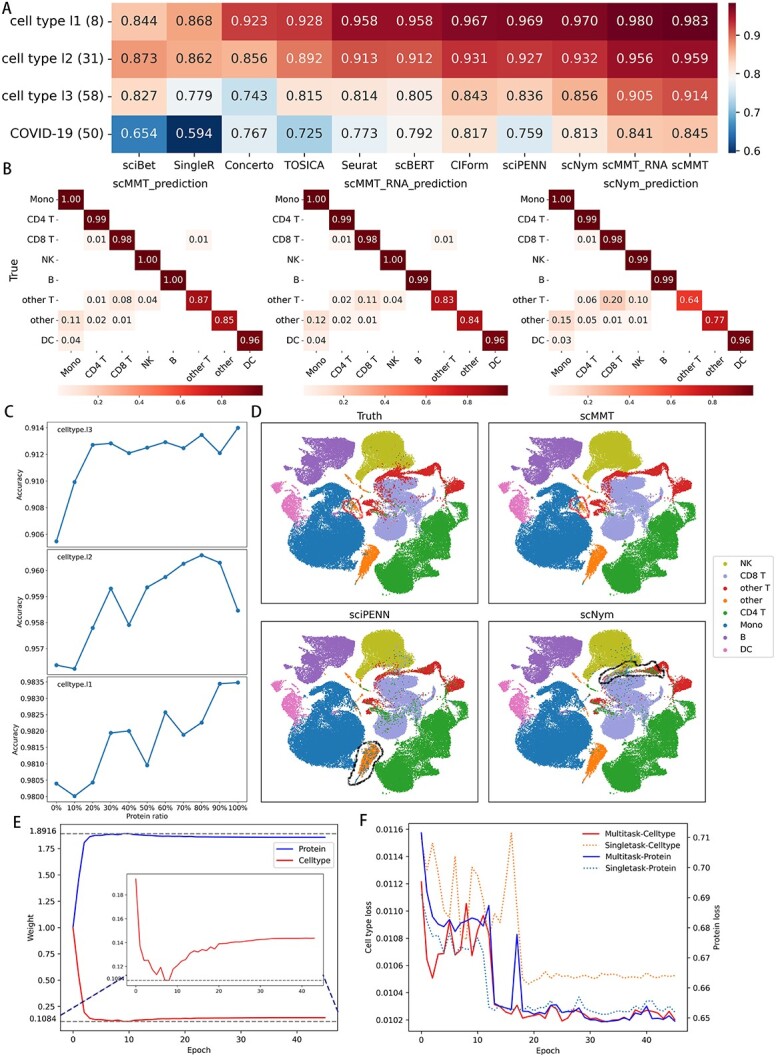
scMMT achieves superior performance in cell type annotation. (**A**) Heatmap depicts the accuracy of cell type annotation. The rows are labeled as cell type l1, cell type l2, cell type l3 and COVID-19 corresponding to the three levels of annotation in the 160k PBMCs dataset and COVID-19 dataset. The numbers in parentheses (8, 31, 58 and 50) indicate the number of cell types in each dataset. (**B**) Heatmaps display the distribution of eight cell type labels predicted by scMMT, scMMT_RNA and scNym (the three methods with the highest accuracy) in the 160k PBMCs dataset. The data were normalized within each row based on the true labels, and only values greater than 0.01 were labeled. (**C**) Line charts depict the impact of protein information proportions on cell type annotation using scMMT. We analyzed three levels of cell annotation in the 160k PBMCs dataset and extracted protein expression data in increments of 10%. (**D**) UMAP plots display the true and predicted eight cell type labels using scMMT, sciPENN and scNym in the 160k PBMCs dataset. Red lines represent cell types that were challenging to identify using other methods but were accurately predicted using scMMT. Conversely, black lines indicate cell types where a specific method had a higher error rate compared to other methods. (**E**) The weight changes of cell type annotation and protein prediction tasks during scMMT training on cell type l3 level using the 160k PBMCs dataset. (**F**) The line graph shows the loss changes of cell type annotation and protein prediction tasks on each epoch of single-task learning and multi-task learning models trained using scMMT on the 160k PBMCs dataset.

Our results demonstrated that both scMMT and scMMT_RNA exhibited higher accuracy in cell type annotation compared to the other methods (Figure 2A). As the number of annotated cell types increased from 8 to 31 and then to 58, the advantages of scMMT/scMMT_RNA over the other methods became increasingly apparent. To assess the performances on various datasets, a validation study was conducted using the COVID-19 dataset. Most methods experienced notable changes in their rankings. However, scMMT and scMMT_RNA still maintained their positions in the first and second places, showcasing their robustness and reliability, with accuracy scores of 0.845 and 0.841, respectively, while most methods were below 0.76.

Next, we evaluated different methods for annotating specific cell types in the 160k PBMCs dataset (Figure 2B, Supplementary Figure S2 available online at http://bib.oxfordjournals.org/). The results showed that scMMT achieved the highest recall rates for all eight cell types of cell type l1, particularly for challenging cell types such as ‘other’ and ‘other T’ cells, which were infrequent and susceptible to being misidentified as other cell types. We also generated UMAP distribution maps to visually compare true cell type labels with predicted labels (Figure 2D, Supplementary Figure S3 available online at http://bib.oxfordjournals.org/). The results showed that scMMT accurately identified rare cell types in specific regions where other methods failed, demonstrating its effectiveness in challenging cell type identification.

To investigate the mutual promotion effect between cell type annotation and protein prediction tasks, we conducted a comparative study using single-task learning and multi-task learning of scMMT. The protein prediction task exhibited a relatively stable decrease in loss compared to the cell type annotation task, quickly gaining significant weight after the initiation of multi-task learning and suppressing error loss induced by a portion of label noise. This facilitated the smooth convergence of the cell type annotation task, ultimately achieving a much lower loss than single-task learning. As the loss of the cell type annotation task stabilized, it also gradually increased its own weight to provide more assistance to the protein prediction task. Both tasks in multi-task learning mutually benefited from each other, resulting in lower final losses compared to single-task learning. Notably, the cell type annotation task, receiving more additional information compared to the protein prediction task, experienced more pronounced advantages in multi-task learning.

To further explore the impact of protein information on scMMT cell type annotation, we conducted an additional analysis using different proportions of proteins at three levels of cell type level in the 160k PBMCs dataset (Figure 2C). The results showed that as the proportion of protein increased, the prediction accuracy for different levels of cell types generally exhibited an upward trend, suggesting that incorporating more protein expression information could enhance the accuracy of cell type annotations. However, occasionally using more protein information resulted in slightly decreased accuracy, probably due to experimental noise and limited predictive utility of certain proteins for cell type identification.

### scMMT enables more accurate identification of rare cell types

**Figure 3:**
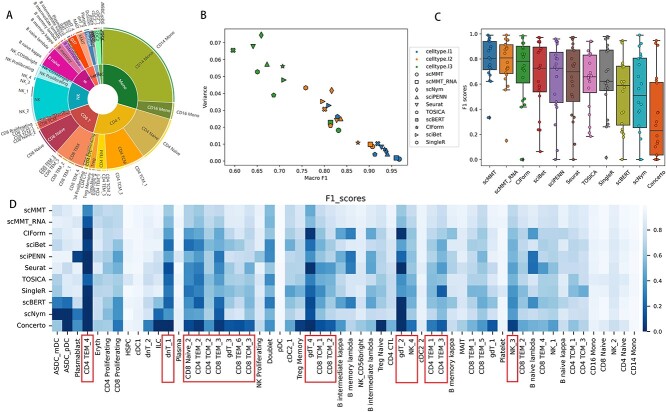
scMMT accurately identifies rare cell types. (**A**) Pie graph displays the distribution of cell types at different hierarchy levels in the 160k PBMCs dataset. The inner circle is composed of 8 cell types from the cell type l1 level, the middle circle is composed of 31 cell types from the cell type l2 level and the outer circle is composed of 58 cell types from the cell type l3 level. (**B**) Scatter plot depicts the distribution of F1 scores for the 10 most effective methods mentioned above. The three distinct colors correspond to three annotation levels, whereas the 10 different shapes represent the 10 methods. (**C**) Box plot displays the distribution of F1 scores for the 20 rarest cell types, which selected from the cell type l3 level of the 160k PBMCs dataset. Each individual point on the plot represents the F1 score for a specific cell type. (**D**) Heatmap displays the F1 scores of the 58 cell types within the cell type l3 level of 160k PBMCs dataset. The heatmap shows a progressive increase in the number of cells from left to right, accompanied by a corresponding trend of increasing F1 scores.

In biological systems, the distribution of cell types is typically uneven. Certain cell types are more abundant, making up a substantial proportion, while others are less common and represent only a small fraction (Figure 3A). As the number of cell types increased, the disparities in abundance between the different cell types became more pronounced. However, rare cell types were also important and sometimes played crucial roles in disease development and progression [49, 50]. Therefore, we evaluated the ability of the various methods to identify rare cell types.

Firstly, we computed the macro F1 scores and variances of different methods for predicting various cell types. The results showed that scMMT consistently exhibited the highest mean scores and the least variation in F1 scores for each level of cell type prediction (Figure 3B, Supplementary Figure S4 available online at http://bib.oxfordjournals.org/). As cell type annotations became more detailed, from the cell type l1 to cell type l3 level, the advantage of scMMT over the other methods was more pronounced. Subsequently, we specifically analyzed the 20 rarest cell types, which comprised only 2.7% of the total cell types within the cell type l3 level. We observed that scMMT and scMMT_RNA exhibited notably higher F1 scores compared to other methods (Figure 3C). Certain cell types, primarily comprising various subtypes of CD4+ T, CD8+ T, NK and other T cells, indicated by red boxes, were challenging to detect using conventional methods (Figure 3D). These cell types often exhibited lower quantities and displayed similarities in RNA expression compared to other cell types [51]. However, scMMT demonstrated efficacy in the identification of these infrequent cell types, thereby underscoring its advantages in discerning rare cell types, particularly those pertaining to immune cells.

### scMMT has the ability to resist dropout and label noise

Owing to the constraints of sequencing technology, low RNA capture rates can lead to the failure of gene expression detection, resulting in a substantial number of random ‘false’ zero count observations. To evaluate the model’s resilience to dropout data, we randomly converted non-zero counts in the CITE-seq simulation dataset to zero in proportion and performed experiments using various methods. To further validate the module’s functionality, we exclusively utilize HVGs in scMMT and scMMT_RNA as controls while ensuring that the input dimensions remain consistent. The results indicate that in the absence of additional dropout noise, the accuracy of various methods is nearly equivalent. The use of multiple feature combinations only resulted in a modest increase of less than 1% in accuracy for the scMMT and scMMT_RNA. Nevertheless, with the escalation of dropout noise proportion, the accuracy of individual models was affected, yet the superiority of employing multiple feature combinations in the scMMT and scMMT_RNA models compared to alternative methods persisted to grow. At 90% dropout noise, the enhancement in accuracy from utilizing multiple feature combinations as opposed to solely using HVGs approached 3%, suggesting the capacity of multiple feature combinations to mitigate dropout noise interference.

**Figure 4:**
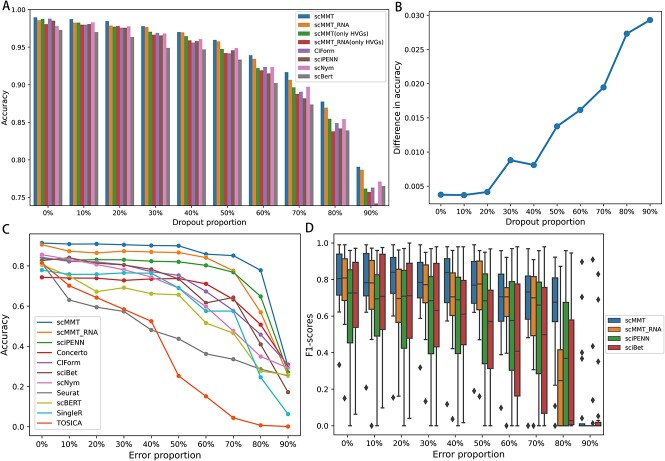
scMMT exhibits robustness against noise arising from incorrect cell type labels. (**A**) Bar chart shows the changes in accuracy of cell type annotation using different methods as additional dropout noise is continuously added to the simulation dataset. (**B**) Line graph illustrates the difference in average accuracy for cell type annotation between scMMT/ scMMT_RNA using multiple feature extraction methods and scMMT(only HVGs)/scMMT_RNA(only HVGs) using only highly variable genes. (**C**) Line graph displays the changes in prediction accuracy for all nine methods as a proportion of erroneous cell type labels was intentionally introduced in cell type l3 level of 160k PBMCs dataset. (**D**) Box plot displays the F1 scores of scMMT, scMMT_RNA, sciPENN and sciBet, which are the top four methods to resist label noise, for annotating rare cell types of cell type l3 level in the 160k PBMCs dataset.

To evaluate the robustness of these methods in resilience to the inherent noise caused by manual annotation [20, 21], we intentionally introduced noise into the annotations of the training dataset using 160k PBMCs dataset. This approach simulated the misclassification scenarios that might arise during manual annotations and was achieved by randomly selecting a specific percentage of cells from each cell type and generating inaccurate cell type labels based on the relative proportions of the remaining cell types. Then, we trained these methods on noisy datasets and evaluated their performances (Figure 4A). The results demonstrated that most methods, such as TOSICA, Seurat and scNym, experienced a rapid decline in the accuracy of cell type prediction as the noise increased. However, scMMT consistently achieved the highest accuracy, maintaining an accuracy of above 0.9 before the noise proportion reached to 50%. Even when the noise proportion reached to 80%, scMMT still achieved an accuracy of approximately 0.8, notably surpassing the other methods. This result indicated that even under challenging annotation conditions, such as extremely low-quality sequencing data and minimal intercellular-type differences, scMMT can still achieve remarkable annotation performance. The notable performance of scMMT under an 80% noise proportion can be attributed to two key reasons. Firstly, the structural design of scMMT exhibited robustness against noise, enabling it to achieve better performance. Secondly, though erroneous labels encompassing 57 different cell types accounted for 80%, the original correct labels constituted the remaining 20% and still made up a large proportion. Additionally, scMMT_RNA maintained its second-place ranking in accuracy until the noise proportion reached 80%. However, it was eventually surpassed by sciPENN, which utilized CITE-seq datasets as reference datasets, similar to scMMT. This indicated that the use of protein expression information from CITE-seq could help correct cell type annotation errors.

To investigate the effect of label noise on the recognition of rare cell types, we used the top four noise-resistant methods mentioned above to conduct an analysis via focusing on the 20 rarest cell types (Figure 4B). The F1 scores of scMMT consistently exhibited the highest values, while the fluctuation of F1 scores for scMMT remained minimal in comparison to other methods, indicating its exceptional stability in recognizing various rare cell types.

### scMMT improves the predictive accuracies in protein expression levels

We evaluated the performance of scMMT in predicting protein expression levels. We conducted two sets of experiments. Firstly, we divided the 160k PBMCs dataset into training and testing sets, focusing on different donors. This dataset consisted of 224 proteins. Secondly, we used the 160k PBMCs dataset as the training set and the H1N1 dataset as the testing set. These two datasets had 59 overlapping proteins. The results revealed that scMMT outperformed the other three methods in terms of the Pearson correlation coefficient and root mean square error for predicting protein expression levels, both within the dataset and across different datasets (Table 2).

**Table 2:** Results of predicting protein expression levels using different methods

Methods	Within 160k PBMCs dataset		160k PBMCs dataset to H1N1 dataset	
	PCC (mean)	RMSE (mean)	PCC (mean)	RMSE (mean)
totalVI	0.451	1.003	0.524	0.925
WNN	0.452	1.095	0.337	1.325
sciPENN	0.489	0.811	0.524	0.793
scMMT	**0.506**	**0.799**	**0.536**	**0.782**

*Note:* Bold fonts indicate the best results.

**Figure 5:**
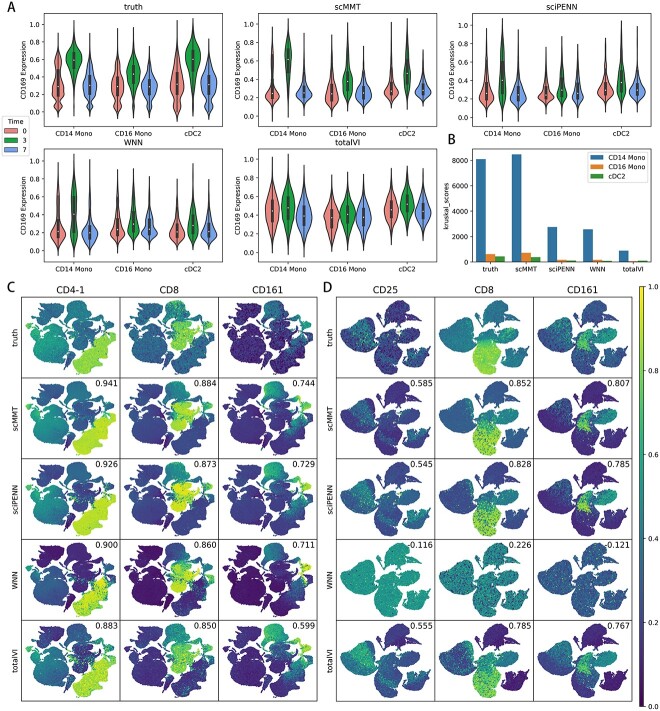
scMMT improves the predictive accuracies in protein expression levels. (**A**) Violin plot displays the feature values of the protein CD169 before (time = 0), 3 days after vaccination (time = 3) and 7 days after vaccination (time = 7) with a VSV-vector HIV vaccine. (**B**) Bar graph displays the Kruskal test values for the distribution differences of CD169 predictions across three cell types and three time periods. (**C**) Feature maps for the proteins CD4-1, CD8 and CD161 were selected from the 160k PBMCs dataset. Each cell in the scatter plot was colored according to the relative intensity of the specified protein value. The numbers in the upper right corner of each image represent the Pearson correlation coefficient between the true and predicted protein expression counts. (**D**) Similar to (C), feature maps for the proteins CD25, CD8 and CD161 were selected from the H1N1 dataset and were used to predict protein expression using the 160k PBMCs dataset.

Next, we tested scMMT’s ability to capture changes in protein expression induced by a vaccine. The 160k PBMCs dataset contained the samples collected from patients before, 3 days after and 7 days after the vaccine was administered. It was reported that CD169 protein in CD14 monocytes, CD16 monocytes and cDC2 cells exhibited a notable response to the vaccine [7]. Specifically, CD169 expression spiked 3 days after vaccination and then returned to their pre-vaccine level after 7 days. This suggested that CD169 is a biomarker for immune response to the vaccine. Therefore, we visualized the CD169 expression in these three cell types at each time point (Figure 5A). For scMMT, a clear peak in predicted CD169 expression was observed 3 days after vaccination in all three cell types, which was consistent with the ground truth. For sciPENN and WNN, the peak was also observed 3 days after vaccination in all three cell types, but less prominently. For totalVI, the peak was not clearly visible. We also conducted a Kruskal–Wallis test [52] to access whether the mean CD169 expression levels were significantly different across the three time points for each method (Figure 5B, Supplementary Table S2 available online at http://bib.oxfordjournals.org/). scMMT achieved the highest Kruskal score among the three cell types, and the predicted results exhibited the smallest deviations from the ground truth. In contrast, sciPENN and WNN exhibited slightly inferior performance, whereas totalVI performed the worst. These results indicated that scMMT had a powerful ability to accurately capture differences across time points and was a valuable tool for identifying stimulus biomarkers.

Moreover, we evaluated the expression of commonly used marker proteins that are widely used to label specific types of cells in biomedical research and clinical applications [53]. In the 160k PBMCs dataset, scMMT consistently exhibited the highest correlation coefficient between the predicted protein expression levels and the actual protein expression levels for nine commonly used marker proteins, including CD4-1, CD8 and CD161 (Figure 5C, Supplementary Figure S5 available online at http://bib.oxfordjournals.org/). We also assessed the transferability of these methods by predicting marker protein expression in the H1N1 dataset using the 160k PBMCs dataset as a reference dataset (Figure 5D, Supplementary Figure S6 available online at http://bib.oxfordjournals.org/). scMMT still showed the highest correlation coefficient and maintained its superior performance. Conversely, WNN exhibited the weakest performance among the evaluated methods. This finding underscored the substantial potential of deep learning approaches in protein expression prediction task.

### scMMT generates a high-quality low-dimensional embedding

**Figure 6:**
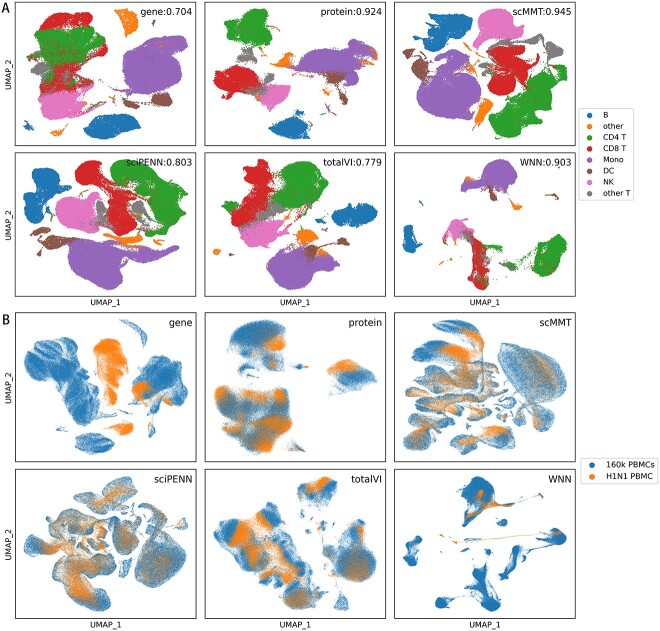
scMMT generates a high-quality low-dimensional embedding representation. (**A**) UMAP distribution plots of the cell type l1 level in the 160k PBMCs dataset for six methods. Cell type l1 level consists of eight different cell types, and each cell type is represented by a different color. The ARI scores are displayed in the upper-left corner, with higher values indicating superior classification performance and closer alignment with the true cell labels. (**B**) UMAP distribution plots display the presence of batch effects when integrating the 160k PBMCs dataset and the H1N1 dataset. Blue points represent cells from the 160k PBMCs dataset; while yellow points represent cells from the H1N1 dataset.

scRNA-seq data are characterized by high dimensionality, noise levels, sparsity and batch effects, making them difficult to visualize and interpret. Therefore, high-quality low-dimensional embedding is crucial for single-cell analyses. To this end, we evaluated the consistency of low-dimensional embedding representations and cell type annotations across different methods. The 160k PBMCs dataset was divided into reference and query subsets based on distinct donors, and the adjusted Rand index (ARI) [54] was used as an evaluation metric (Figure 6A). The results indicated that the low-dimensional embedding representation of scMMT exhibited the highest capability in distinguishing different cell types, achieving an impressive ARI score of 0.945. Representations that utilized only protein information closely followed scMMT and ranked second in performance. However, the representation relying solely on scRNA-seq data performed the poorest outcomes. This limitation reflected the inherent challenge of using scRNA-seq data to distinguish cells with similar structures but varying functions, particularly CD8+ T, CD4+ T and other T cells. The ARI scores of WNN, sciPENN and totalVI fell between the scores achieved using only protein data and that using only scRNA-seq data. This can be attributed to their limited ability to effectively capture the relationship between RNA and protein.

Next, to further explore the representation of rare cell types, we focused on ‘other T’ cells in cell type l1 level, which encompassed seven cell subtypes and were challenging to distinguish due to their small quantities and easy confusion. Notably, scMMT exhibited distinct boundary segmentation and minimal overlap between different subpopulations, maintaining its position as the best method with an impressive ARI value of 0.459 (Supplementary Figure S7 available online at http://bib.oxfordjournals.org/). In contrast, the other methods were more prone to overlap among different subpopulations.

Finally, we examined the ability of different methods to address batch effects. We used 160k PBMCs and H1N1 datasets as reference and query datasets, respectively. The results revealed that scMMT, sciPENN and totalVI generated low-dimensional embeddings that exhibited a uniform mixing of the two datasets (Figure 6B), indicating their effectiveness in mitigating cross-dataset batch effects. Conversely, WNN performed poorly in this regard. Additionally, the representation using only protein data outperformed that using only scRNA-seq data. This finding suggested that deep learning methods provide a considerable advantage in understanding the complex relationship between RNA and protein and mitigating batch effects.

## DISCUSSION

This paper proposes a novel method, scMMT, aimed at improving the precision of cell annotation, protein prediction and low-dimensional embedding. The key contributions of this approach encompass the proposal of a new feature extraction method that integrates TSVD, FA, ComBat and HVGs, addressing the limitations of existing techniques reliant solely on HVGs. Additionally, the incorporation of multi-task learning based on the GradNorm method establishes linkages between cell type annotation and protein prediction tasks, resulting in mutually enhanced outcomes. Furthermore, the introduction of logarithmic weighting and label smoothing mechanisms serves to bolster the recognition capability of rare cell types and mitigate model overconfidence.

Through ablation experiments and comparisons with existing methods, we found that our proposed feature extraction method and multi-task learning framework excelled in combating dropout and cell label noise and achieved state-of-the-art performance in cell type annotation, rare cell identification, dropout and label noise resistance, protein expression prediction and low-dimensional embedding representation. Particularly, scMMT demonstrated remarkable ability in identifying immune cell types with similar molecular profiles but distinct functions, as well as capturing changes in protein expression induced by a vaccine. This exceptional capability of scMMT holds great promise in advancing immunological research by providing valuable insights and assistance.

Additionally, scMMT exhibited exceptional flexibility by supporting the utilization of both scRNA-seq and CITE-seq datasets as reference datasets. This allowed researchers to leverage diverse data types and further expand the applicability of scMMT to a wide range of research fields. Finally, scMMT demonstrated low time complexity compared to other tools with similar functionalities (Supplementary Figure S8 available online at http://bib.oxfordjournals.org/). Overall, the exceptional accuracy, robustness, flexibility and low time complexity make scMMT an efficient and practical tool for large-scale data analysis.

With the rapid development of single-cell multi-modal sequencing technology, an increasing number of modalities is becoming available for single cells. Here, we utilized only the protein and RNA information obtained through CITE-seq data to establish the model for single-cell analysis. If other modalities, particularly spatial position information, can be added to the model, its performance could be further enhanced.

**Key Points**

- We proposed an efficient feature extraction method that integrates TSVD, FA, ComBat and HVGs to effectively capture comprehensive information from high-dimensional and sparse scRNA-seq data.
- We utilized CITE-seq datasets as reference datasets to overcome the limitations of scRNA-seq data in terms of the lack of functional information and constructed a multi-task learning model based on the GradNorm method to facilitate mutual reinforcement between cell type annotation and protein prediction tasks.
- We employed logarithmic weighting and label smoothing mechanism to mitigate the challenges posed by imbalanced and noisy cell type labels.
- scMMT has shown outstanding performance in various aspects. Particularly, it excels in identifying immune cell types with similar molecular profiles but distinct functions and capturing changes in protein expression induced by vaccination, which makes scMMT a valuable tool for advancing immunological research.

## Supplementary Material

Supplementary_Materials_bbad523

## Data Availability

We analyzed multiple published CITE-seq datasets throughout the evaluations. These data are available as follows (accession numbers provided where possible). (1) Seurat 4 human peripheral blood mononuclear cells dataset (GEO: GSE164378). (2) H1N1 influenza PBMCs dataset (https://doi.org/10.35092/yhjc.c.4753772). (3) Haniffa COVID-19 dataset (https://www.ebi.ac.uk/arrayexpress/experiments/E-MTAB-10026/). (4) Simulation dataset (https://github.com/SongqiZhou/scMMT/releases/tag/scMMT).

## CODE AVAILABILITY

Software is available at “scMMT [https://github.com/SongqiZhou/scMMT]”.

## Author Biographies

**Songqi Zhou** is a master’s student of Chongqing Institute of Green and Intelligent Technology, Chinese Academy of Sciences. His research focuses on computational methods and tools development for biological data analysis.

**Yang Li** is a researcher of Chongqing Research Institute of Big Data, Peking University. His research interest is host response to infection based on multi-omics data.

**Wenyuan Wu** is a professor of Chongqing Institute of Green and Intelligent Technology, Chinese Academy of Sciences. His research focuses on mathematical models and computational methods for biological data analysis.

**Li Li** is a professor of Chongqing Institute of Green and Intelligent Technology, Chinese Academy of Sciences. His research focuses on bioinformatics in organ development.
