# Supplementary material for: scMMT: a multi-use deep learning approach for cell annotation, protein prediction and embedding in single-cell RNA-seq data
Source: Brief Bioinform. 2024 Jan 31;25(2):bbad523. doi: 10.1093/bib/bbad523 (PMC10833085; doi:10.1093/bib/bbad523)
Supplement: Supplementary_Materials_bbad523 [file supplementary_materials_bbad523.docx]

**scMMT: A Multi-use Deep Learning Approach for Cell Annotation, Protein Prediction, and Embedding in Single-cell RNA-seq Data**

Songqi Zhou^1,2^, Yang Li^1,2,3^, Wenyuan Wu^1,2*^ and Li Li^1,2*^

^1^Chongqing Institute of Green and Intelligent Technology, Chinese Academy of Sciences, 400714, P.R. China

^2^Chongqing School, University of Chinese Academy of Sciences, Chongqing, China

^3^Chongqing Research Institute of Big Data, Peking University, Chongqing, China

^*^Corresponding author. E-mail: lili@cigit.ac.cn (L. L), wuwenyuan@cigit.ac.cn (W. W)

**Supplementary Materials**

**Tables**

**Supplementary Table S1.** The sizes of the reference dataset and query dataset in each experiment

| Section | Reference dataset | Reference dataset’s size | Query dataset | Query dataset’s size |
| --- | --- | --- | --- | --- |
| 1. scMMT enables more accurate cell type annotation | 160k PBMCs dataset (donor 1,3,4,7) | 75,756 | 160k PBMCs dataset (donor 2,5,6,8) | 86,008 |
|  | COVID-19 dataset (batch1*) | 177,377 | COVID-19 dataset (batch2*) | 103,045 |
| 2.scMMT enables more accurate identification of rare cell types | 160k PBMCs dataset (donor 1,3,4,7) | 75,756 | 160k PBMCs dataset (donor 2,5,6,8) | 86,008 |
| 3.scMMT has the ability to resist dropout and label noise | Simulation dataset (Stratified Shuffle Split) | 10,000 | Simulation dataset | 10,000 |
|  | 160k PBMCs dataset (donor 1,3,4,7) | 75,756 | 160k PBMCs dataset (donor 2,5,6,8) | 86,008 |
| 4.scMMT improves the predictive accuracies in protein expression levels | 160k PBMCs dataset (donor 1,3,4,7) | 75,756 | 160k PBMCs dataset (donor 2,5,6,8) | 86,008 |
|  | 160k PBMCs dataset | 161,764 | H1N1 dataset | 53,201 |
| 5.scMMT generates a high-quality low-dimensional embedding | 160k PBMCs dataset (donor 1,3,4,7) | 75,756 | 160k PBMCs dataset (donor 2,5,6,8) | 86,008 |

Note: Batch1* contains samples: 'MH9143427', 'AP11', 'MH9143326', 'MH9143270', 'MH9143324', 'MH9143422', 'MH9143271', 'MH9143423', 'MH9143325', 'MH9179825', 'MH8919327', 'MH9143321', 'AP12', 'MH9143424', 'newcastle49', 'MH9179824', 'MH8919328', 'MH9143421', 'MH8919332'; Batch2* contains samples: 'AP6', 'MH8919333', 'MH9143277', 'MH9143327', 'MH9143276', MH9143323', 'MH9143272', 'AP5', 'MH9143275', 'MH9143322'.

**Supplementary Table S2.** Results of Kruskal-Wallis test

| Methods | CD14 | |  | CD16 | |  | cDC2 | |
| --- | --- | --- | --- | --- | --- | --- | --- | --- |
|  | Kruskal score | p value |  | Kruskal score | p value |  | Kruskal score | p value |
| Truth | 8097.250 | 0 |  | 614.539 | 3.585E-134 |  | 433.856 | 6.159E-95 |
| totalVI | 887.057 | 2.387E-193 |  | 68.602 | 1.269E-15 |  | 98.892 | 3.356E-22 |
| WNN | 2573.542 | 0 |  | 161.724 | 7.622E-36 |  | 79.071 | 6.760E-18 |
| sciPENN | 2748.362 | 0 |  | 165.803 | 9.918E-37 |  | 112.749 | 3.288E-25 |
| scMMT | 8470.914 | 0 |  | 714.536 | 6.926E-156 |  | 379.621 | 3.685E-83 |

**Figures**


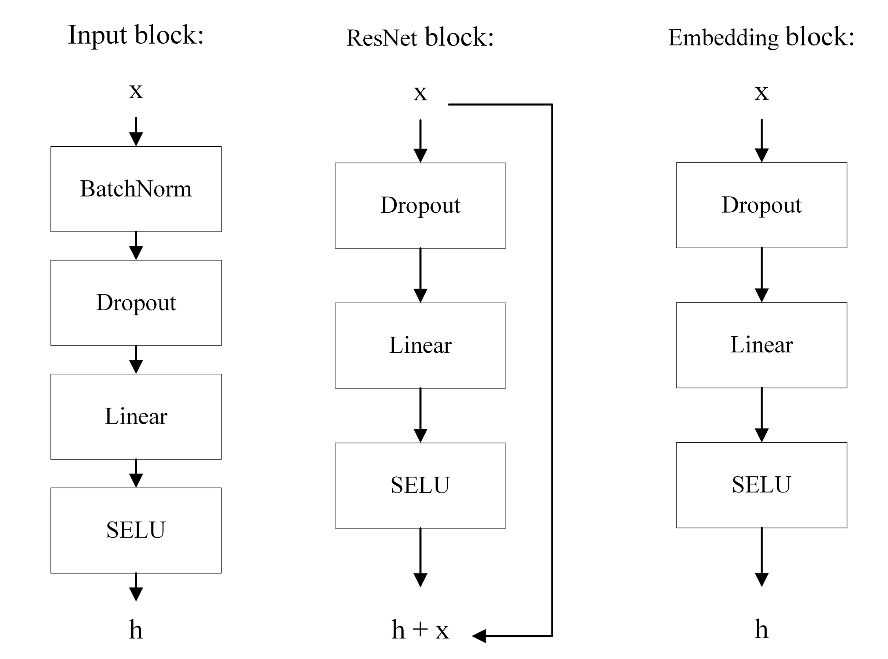


**Supplementary Figure S1. Structure diagrams for the Input block, ResNet block, and Embedding block.** Specifically, the input block consists of BatchNorm, Dropout, Linear, and SELU layers. The ResNet block and Embedding block consist of Dropout, Linear, and SELU layers.


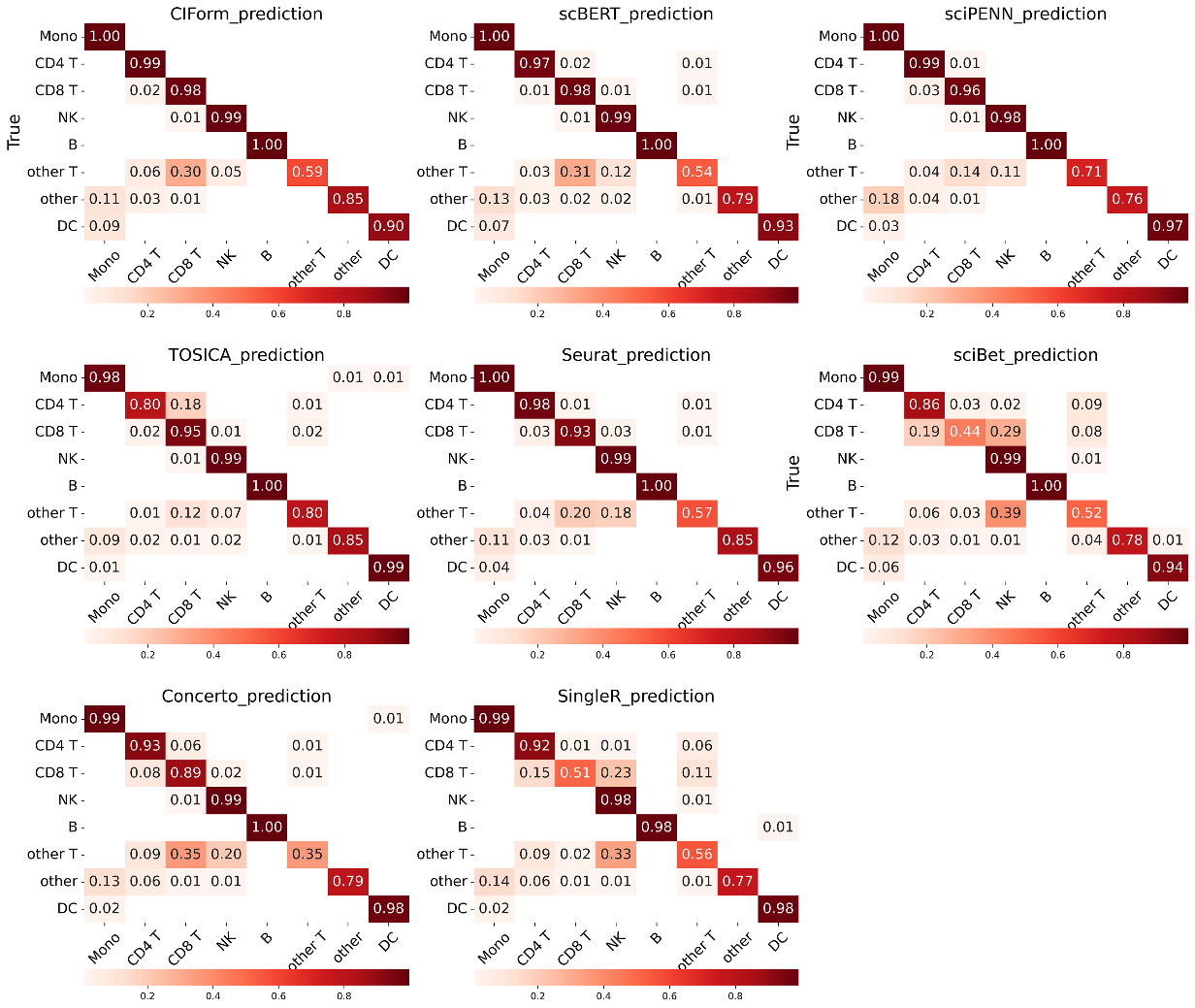


**Supplementary Figure S2.** **Prediction of 8 cell types using 8 methods.** Heatmaps display the distributions of cell-type labels predicted by CIForm, scBERT, sciPENN, TOSICA, Seurat, sciBet, Concerto and SingleR for the cell type l1 level in the 160k PBMCs dataset. The horizontal axis represents the predicted label, while the vertical axis shows the true label. The data were normalized within each row based on the true label, and only values greater than 0.01 were labeled.


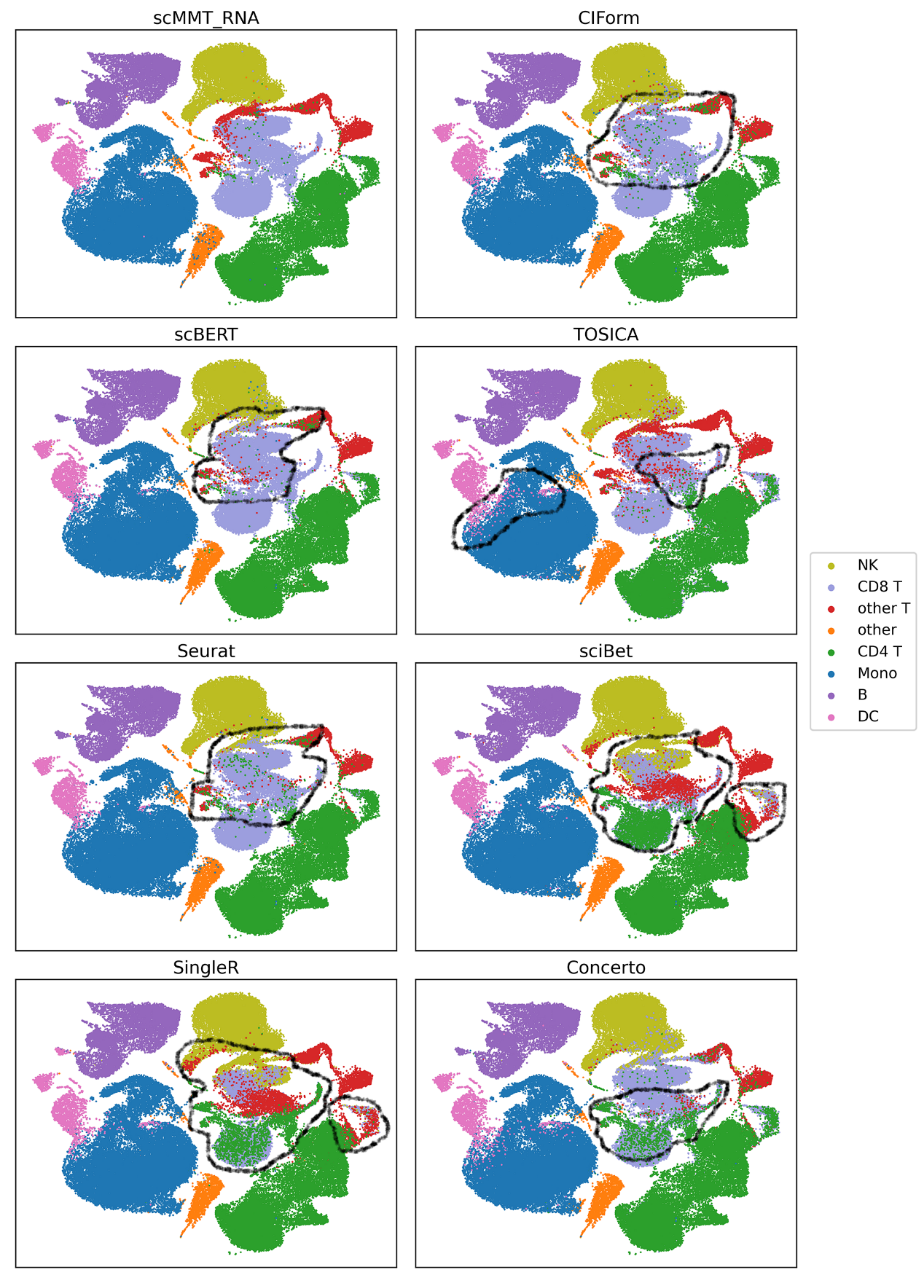


**Supplementary Figure S3. Predictions of UMAP distribution for 8 cell types using 8 methods.** UMAP plots depict the true and predicted cell-type labels for cell type l1 level in the 160k PBMCs dataset using scMMT_RNA, CIForm, scBERT, TOSICA, Seurat, sciBet, SingleR and Concerto, respectively. The black dashed lines indicate regions where a particular method had a higher error rate compared to other methods.


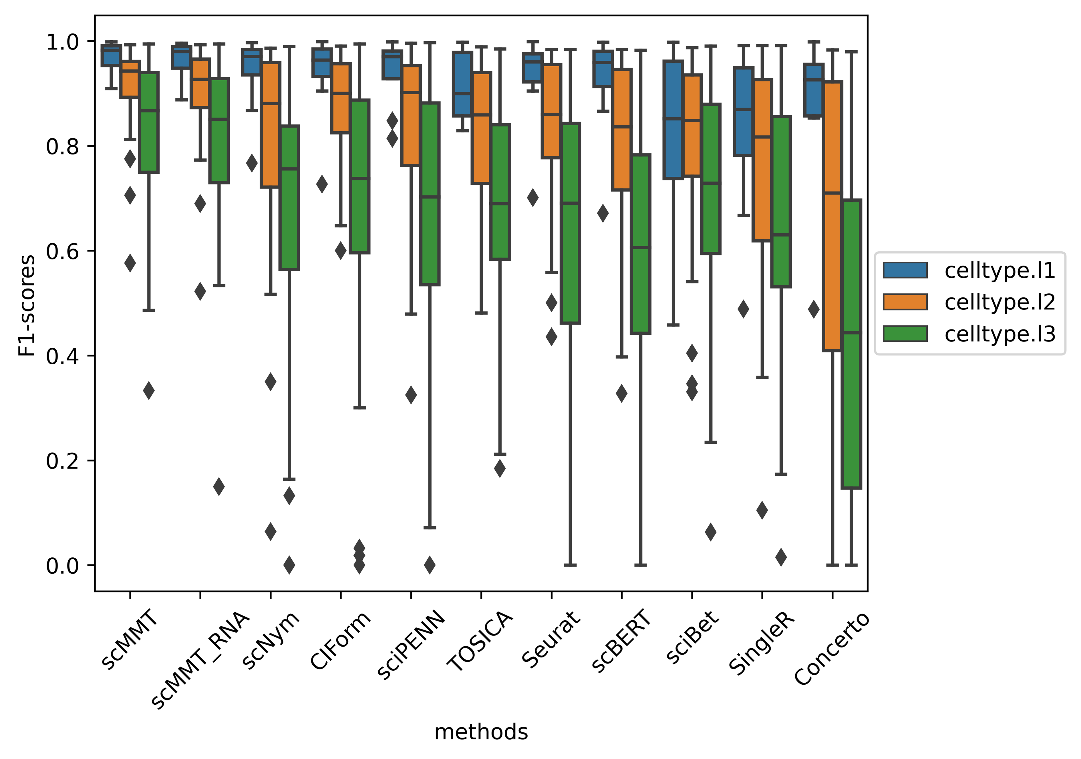


**Supplementary Figure S4. scMMT and scMMT_RNA outperformed the other methods in F1 scores.** Box plot displays the distribution of F1 scores for the three levels of annotation in the 160k PBMCs dataset. Eleven methods were employed to predict cell types, with different colors representing predictions of different levels. Among the methods considered, scMMT and scMMT_RNA exhibited superior performance in terms of F1 scores across all three levels. They displayed the highest median F1 scores and the least variability.


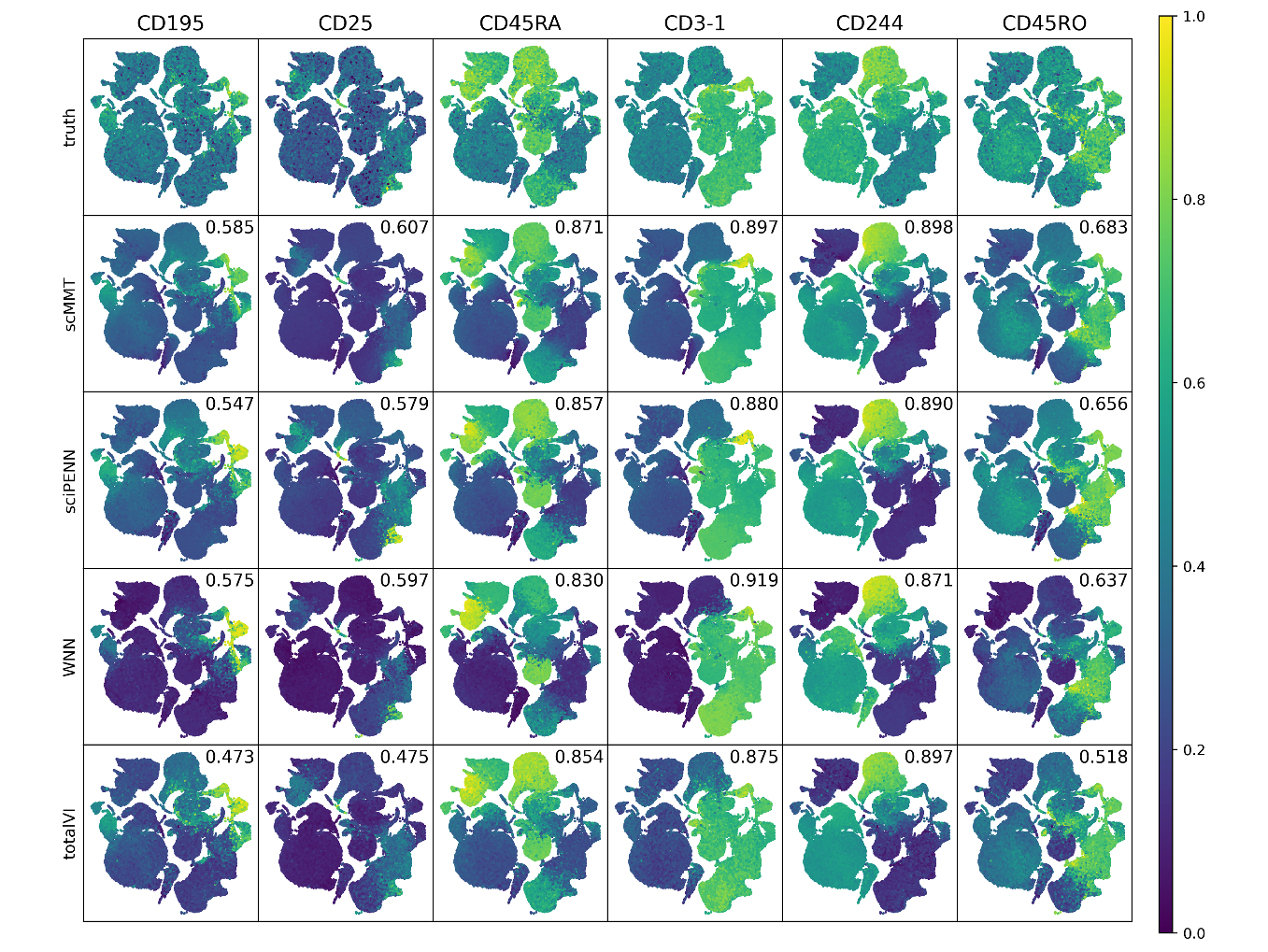


**Supplementary Figure S5. Feature maps for the proteins CD195, CD25, CD45RA, CD3-1, CD244, and CD45RO.** The proteins were selected from the 160k PBMCs dataset. Each cell in the scatter plots was colored according to the relative intensity of the specified protein value. In the first row, the true values were used to guide the color mapping of the feature maps. In the subsequent rows, the protein expression of each cell was colored based on the predicted results from scMMT, sciPENN, WNN, and totalVI. The numbers in the upper right corner of each image represent the Pearson correlation coefficient between the true and predicted protein expression counts.


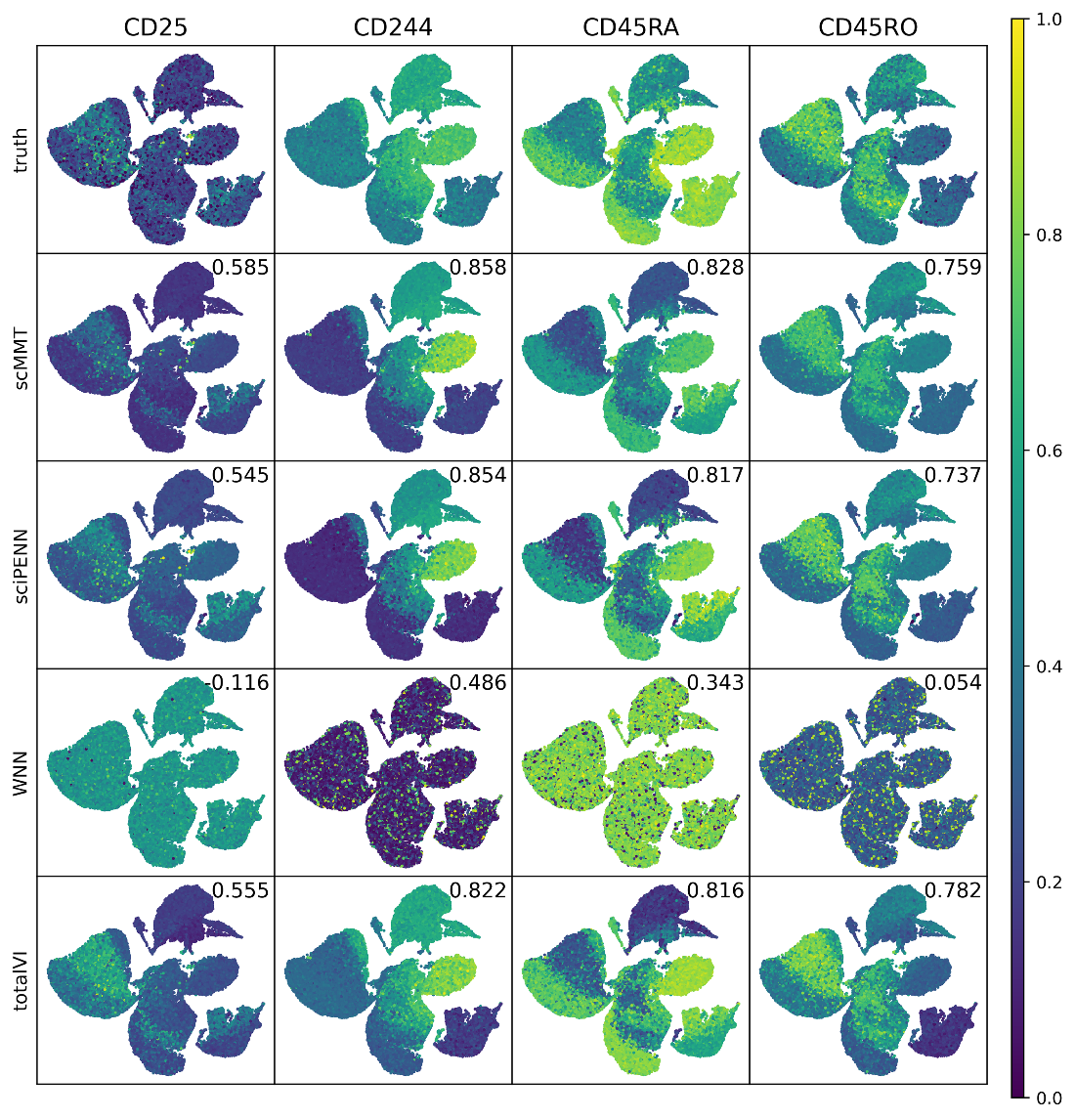


**Supplementary Figure S6. Feature maps for the proteins CD195, CD244, CD45RA and CD45RO.** The proteins were selected from overlapping parts of the 160k PBMCs dataset and H1N1 dataset. Each cell in the scatter plot was colored according to the relative intensity of the specified protein value. In the first row, the true values were used to guide the color mapping of the feature maps. In the subsequent rows, the protein expression of each cell was colored based on the predicted results from scMMT, sciPENN, WNN, and totalVI. The numbers in the upper right corner of each image represent the Pearson correlation coefficient between the true and predicted protein expression counts.


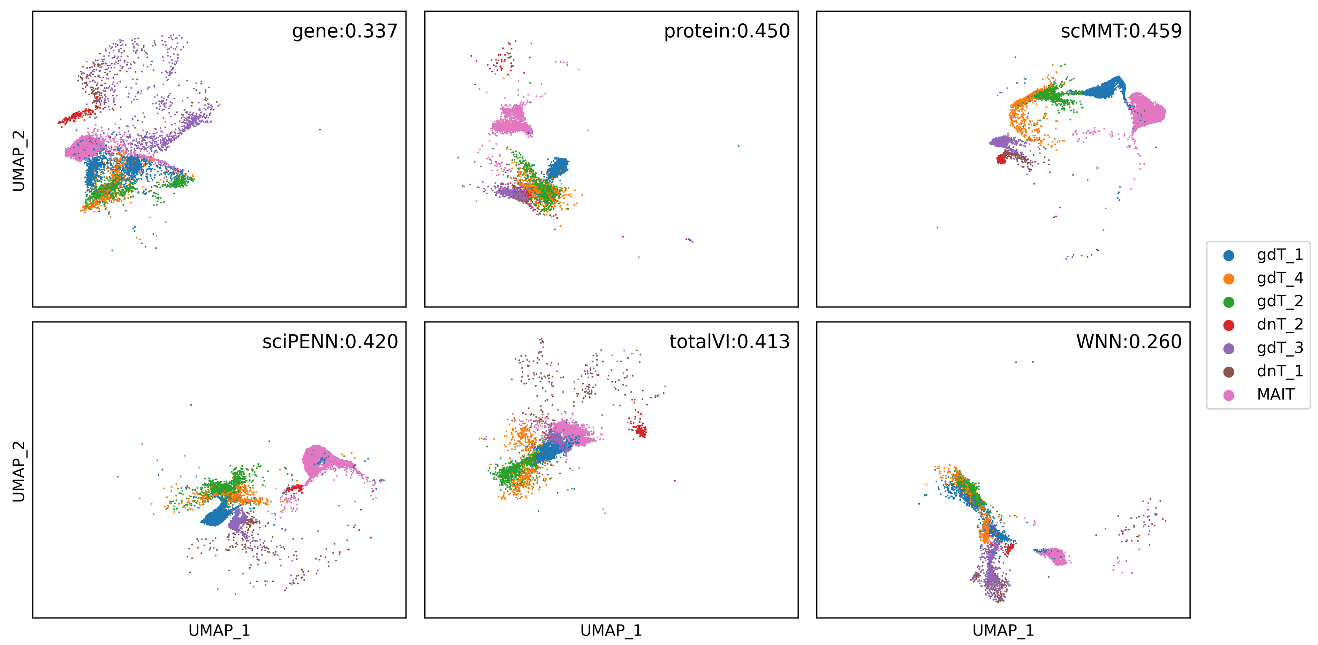


**Supplementary Figure S7.** **UMAP distribution plots of “other T” cells in cell type l1 level of the 160k PBMCs dataset for six methods.** “other T” cells consists of seven different cell types, and each cell type is represented by a different color. The ARI scores are displayed in the upper left corner, with higher values indicating superior classification performance and closer alignment with the true cell labels.


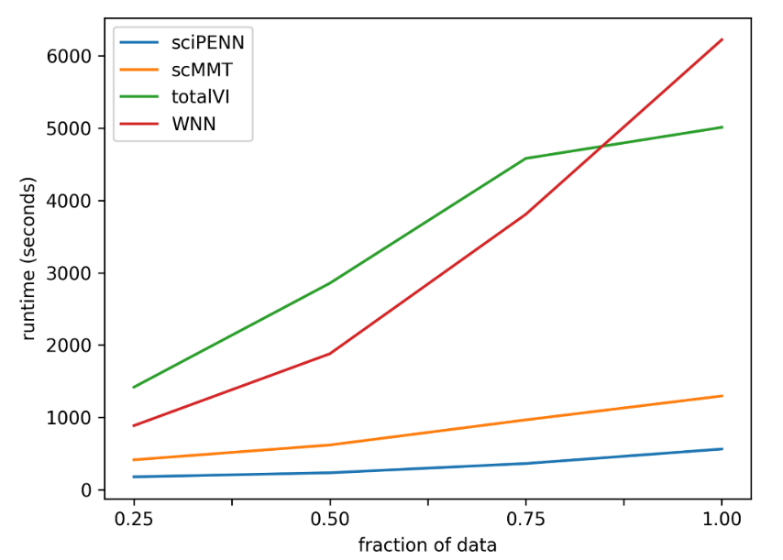


**Supplementary Figure S8. Runtime comparison of methods.** This figure showcases the computational efficiency of scMMT compared to WNN, totalVI, and sciPENN. Specifically, we conducted experiments using the 160k PBMCs dataset consisting of 161,746 cells as the reference data, and the H1N1 dataset comprising 53,201 cells as the query data. Our approach involved training each method using a fraction of the 160k PBMCs dataset (corresponding to 100*f% of the data) and generating predictions on the same fraction of the query data. We recorded the time taken for this process across various fractions for each method. The resulting timings were then plotted to visualize the computational performance of each method. All experiments were conducted on a single server with 1T of memory, an Intel(R) Xeon(R) Platinum 8369B CPU @ 2.90GHz with 128 threads, and a 4070Ti graphics processing unit (GPU).
